# Outbreak of Cyclosporiasis Associated with Imported Raspberries, Philadelphia, Pennsylvania, 2000

**DOI:** 10.3201/eid0808.020012

**Published:** 2002-08

**Authors:** Alice Y. Ho, Adriana S. Lopez, Michael G. Eberhart, Robert Levenson, Bernard S. Finkel, Alexandre J. da Silva, Jacquelin M. Roberts, Palmer A. Orlandi, Caroline C. Johnson, Barbara L. Herwaldt

**Affiliations:** * Philadelphia Department of Public Health, Philadelphia, Pennsylvania, USA; † Atlanta Research and Education Foundation, Atlanta, Georgia, USA; ‡ Centers for Disease Control and Prevention, Atlanta, Georgia, USA; § Food and Drug Administration, Washington, DC, USA

**Keywords:** *Cyclospora cayetanensis*, cyclosporiasis, raspberries, Guatemala, Pennsylvania

## Abstract

An outbreak of cyclosporiasis occurred in attendees of a wedding reception held in Philadelphia, Pennsylvania, on June 10, 2000. In a retrospective cohort study, 54 (68.4%) of the 79 interviewed guests and members of the wedding party met the case definition. The wedding cake, which had a cream filling that included raspberries, was the food item most strongly associated with illness (multivariate relative risk, 5.9; 95% confidence interval, 3.6 to 10.5). Leftover cake was positive for *Cyclospora* DNA by polymerase chain reaction analyses. Sequencing of the amplified fragments confirmed that the organism was *Cyclospora cayetanensis*. The year 2000 was the fifth year since 1995 that outbreaks of cyclosporiasis definitely or probably associated with Guatemalan raspberries have occurred in the spring in North America. Additionally, this is the second documented U.S. outbreak, and the first associated with raspberries, for which *Cyclospora* has been detected in the epidemiologically implicated food item.

On June 10, 2000, a total of 83 persons attended a catered wedding reception in Pennsylvania. Approximately 8 days later, the bride notified the local health department that she, her husband, and many guests at the reception had a gastrointestinal illness. Stool specimens from attendees were positive for oocysts of the coccidian parasite *Cyclospora cayetanensis*, and an epidemiologic investigation was begun by the Philadelphia Department of Public Health and the Centers for Disease Control and Prevention (CDC).

 Previous foodborne outbreaks of cyclosporiasis in the United States have been associated with consumption of fresh raspberries, mesclun lettuce, and basil (1–8). An important feature of the biology of *Cyclospora* is that oocysts excreted in feces require days to weeks outside the host to sporulate and thus to become infectious. However, the minimum time required for sporulation is unknown. *Cyclospora* oocysts must survive in the environment long enough to sporulate and infect a susceptible host, which suggests that they are very hardy.

## Methods

### Epidemiologic Investigation

 A retrospective cohort study was conducted by administering a structured questionnaire by telephone about symptoms and event-related exposures. A case of cyclosporiasis was defined as onset of illness 1–14 days after the reception and either (a) one positive stool specimen and at least one gastrointestinal symptom (i.e., loose stools, nausea, vomiting, stomach cramps, gas/bloating, loss of appetite, and unintentional weight loss) or constitutional symptom (i.e., fever, chills, muscle aches, generalized body aches, and headaches); (b) any loose stools in a 24-hour period and at least one other gastrointestinal or constitutional symptom; or (c) a total of at least three gastrointestinal symptoms.

 We attempted to identify more cases of cyclosporiasis by having the Pennsylvania State Bureau of Epidemiology alert health departments throughout the state of the investigation. We also requested the catering company to contact the six groups to which they had served raspberries during the period June 2–4 that might have been from the suspected raspberry shipments.

### Statistical Analyses

 Univariate relative risks and 95% confidence intervals (CIs) were calculated by using Epi-Info, version 6.04 (CDC). Multivariate Poisson regression analyses were conducted by using SAS, version 8.0 for Windows (SAS Institute Inc., Cary, NC).

### Laboratory Investigation<H2>

The local diagnostic reference laboratory examined stool specimens from five patients for *Cyclospora* oocysts by Kinyoun’s modified acid-fast technique. CDC reevaluated specimens from three of these patients either by examining slides made by the local laboratory or by UV fluorescence microscopy of a stool specimen concentrated by the formalin-ethyl acetate technique. An unknown number of specimens were tested at the local diagnostic reference laboratory for *Cryptosporidium parvum*; *Salmonella*, *Shigella*, and *Campylobacter* spp.; and *Escherichia coli* O157:H7.

 CDC and the Food and Drug Administration (FDA) used molecular methods to examine leftover wedding cake, which the bride and groom had frozen, for *Cyclospora*. FDA’s methods were as follows. One-gram portions of the icing from the top of the wedding cake, the raspberry filling, and the cake itself were processed for molecular analyses by using 20 mL of Tris buffer (containing 100 mM Tris-HCL, pH 8.0, 10 mM EDTA, 150 mM NaCl) and 5 mL of ethyl acetate. The samples were shaken vigorously, heated at 56°C, and gently agitated for 30 min. The samples were centrifuged for 15 min at 4500 x *g* at 15°C. The organic phase, the interface, and the aqueous (upper) phase were aspirated. Next, the pellet was washed twice with 25 mL of Tris buffer and centrifuged as above. The pellet was suspended in 4 mL of Tris buffer, layered over a 4-mL Optiprep solution (Invitrogen Corporation, Carlsbad, CA), and diluted 1:1 with the Tris buffer. The samples were centrifuged for 15 min at 250 x *g* at 15°C. The aqueous phase, the interface, and 1.5 mL of the upper portion of the Optiprep layer were removed and saved in a 15-mL centrifuge tube. The remaining pellet and Optiprep solution were discarded. All saved samples were diluted further with 2–3 mL of Tris buffer and centrifuged for 10 min at 20,000 x *g.*

The supernatant from each sample was aspirated to within 1 mL of the bottom, and the pellets were suspended in this volume and transferred to a 1.5-mL microcentrifuge tube. The samples were then centrifuged for 2 min in a microcentrifuge at 14,000 x *g,* and the supernatant was aspirated. The final pellets were suspended in 50 µL of distilled water, and a 10-µL volume of each sample was spotted onto Flinders Technologies of Australia (FTA) filters (Fitzco, Inc., Maple Plain, MN) for DNA extraction and subsequent polymerase chain reaction (PCR) analyses as described previously (9).

CDC’s methods were as follows. DNA was extracted directly from nine randomly selected 1-g portions of raspberry filling from the cake by using selected components from the FastDNA kit (BIO 101, Inc., Vista, CA). The portions were placed into tubes containing disruption beads that were included in the kit, and DNA extraction was performed as described (10).

Nested PCR amplification was performed by using primers modified from those described elsewhere (11). The restriction sites *Eco*RI and *Bam*HI were removed from the 5´ end, and bases matching the *C. cayetanensis* small subunit (SSU) rRNA coding region were added. Thus, primers CYCF1 (5´-ATTACCCAATGAAAACAGTTT-3´) and CYCR2 (5´-TGCAGGAGAAGCCAAGGTAGG-3´) were used for the first step of nested amplification; the preamplified fragment was nested with primers CYCF3 (5´-GCCTTCCGCGCTTCGCTGCGT-3´) and CYCR4 (5´- TCGTCTTCAAACCCCCTACTG-3´). Amplification with these primers generates a 294-bp fragment from the *C. cayetanensis* SSUrRNA coding region.

Besides the nested PCR, a 636-bp fragment from the *Cyclospora* sp. SSUrRNA, flanked by primers CYCF1/CYCR2, was amplified and sequenced to identify the parasite at the species level. This fragment contains substitutions that allow differentiation among the *Cyclospora* spp. for which the sequence of the SSUrRNA coding region is available in GenBank (i.e., *C. cayetanensis*, *C. cercopitheci*, *C. colobi*, and *C. papionis*). Sequencing reactions were performed on PCR products that had been purified with the StrataPrep PCR Purification Kit (Stratagene, La Jolla, CA). Sequencing reactions, analyses, and assemblage of sequences were performed as described (12).

### Environmental and Source Investigations

 The reception facility was inspected, and food-handling and storage practices were observed. Information about illness in the catering staff was collected by examining the caterer’s sick logs. The caterer was also asked to contact the health department if staff called in sick. Ill staff members would then be asked whether they had had a gastrointestinal illness during the 3 weeks before and after the reception.

 The caterer and the distributors that supplied his company were interviewed, and shipping documents were obtained to determine the source of the raspberries used in the cake (13). In cooperation with the Guatemalan government, FDA inspected one of the farms that could have supplied the raspberries served at the wedding; this farm also could have supplied the raspberries for an event in another state in 2000 that was associated with an outbreak of cyclosporiasis.

## Results

### Epidemiologic Investigation

**Figure 1:**
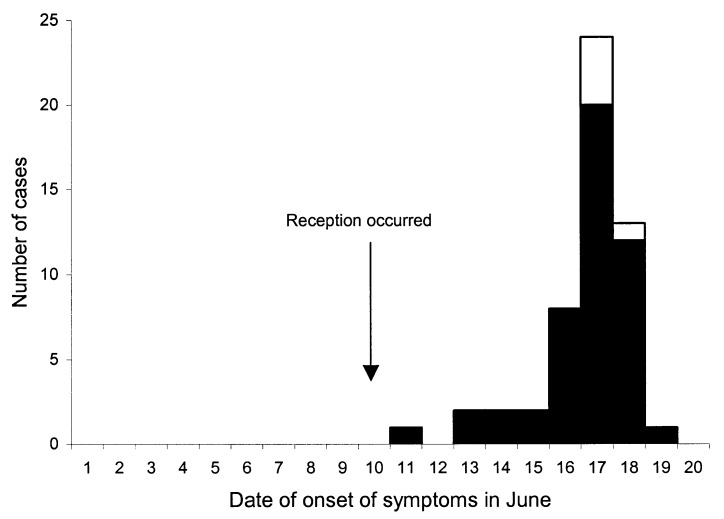
Dates of onset of symptoms in laboratory-confirmed case-patients (white box; n = 5) and clinically defined case-patients (black box; n = 49) who attended the wedding reception in June 2000 in Pennsylvania. The exact date of onset of symptoms was not specified for one case-patient who became ill after the reception.

 Seventy-nine (95.2%) of the 83 attendees of the wedding reception were interviewed. Fifty-four (68.4%) of the 79 had illness that met the case definition. Five (9.3%) of the 54 case-patients had laboratory-confirmed cyclosporiasis, and 49 (90.7%) of the 54 case-patients had clinically defined cyclosporiasis. All case-patients who met part (c) of the case definition also met part (b). Therefore, parts (b) and (c) were combined to represent the clinically defined category of the case definition. The median incubation period was 7 days (range 1–9 days) (Figure 1). The symptoms associated with illness are listed in Table 1.

**Table 1:** Information on attendees of a wedding reception associated with an outbreak of cyclosporiasis, Pennsylvania, June 2000

Variable	Case-patients (n=54)^a^	Non-case attendees (n=25)^b^
Age, y, median (range)^c^	38.8 (18–87)	
Female sex, no. (%)^c^	32 (59.3)	
Symptoms, no. (%)		
Diarrhea^d^	54 (100)	1 (4.0)
Nausea	43 (79.6)	1 (4.0)
Stomach cramps	41 (75.9)	1 (4.0)
Chills/sweats	41 (75.9)	0
Headache	36 (66.7)	0
Muscle/body aches	36 (66.7)	1 (4.0)
Fever^e^	32 (59.3)	0
Vomiting	26 (48.1)	0
Dizziness	25 (46.3)	0
Rash	3 (5.6)	0
Hospitalization, no. (%)	2 (3.7)	0
Length of stay, days, median (range)	12 (10–14)	0

^a^ Five of the 54 case-patients had laboratory-confirmed cyclosporiasis. ^b^Two of the 25 non-case attendees reported having at least one symptom. ^c^Information about age and sex was obtained from only two non-case attendees and was not included in the table. ^d^Diarrhea was defined as having three or more loose stools in a 24-hour period. ^e^In case-patients, the median temperature was 37.8°C (range 37.2–38.5°C). No non-case attendees were febrile.

 Several food items were significantly associated with illness in univariate analyses (Table 2). However, only the cake, which had a cream filling that included pieces of raspberries, was significantly associated with illness in multivariate analyses (relative risk 5.9; 95% CI 3.6 to 10.5). Mesclun lettuce was used as a garnish for several food items, and fresh basil was served in one food item; neither was significantly associated with illness.

**Table 2:** Food items at a wedding reception that were significantly associated with cyclosporiasis in univariate analyses, Pennsylvania, June 2000

	Attack rate in attendees, no. ill/no. exposed or unexposed (%)			
Food items^a^	Exposed		Unexposed	RR (95% CI)
Wedding cake^b^	50/53 (94.3)	4/26 (15.4)		6.1 (2.5 to 15.1)
Fresh fruit^c^	36/43 (83.7)	18/36 (50.0)		1.7 (1.2 to 2.4)
Arugula salad	34/41 (82.9)	20/38 (52.6)		1.6 (1.1 to 2.2)
Focaccia bread	15/16 (93.8)	39/63 (61.9)		1.5 (1.2 to 1.9)
Hearthbaked bread	23/27 (85.2)	31/52 (59.6)		1.4 (1.1 to 1.9)

^a^Mesclun lettuce was served as a garnish on several hors d’oeuvres trays, and basil was served fresh in one food item. Neither was significantly associated with illness. ^b^Food item was statistically significant in multivariate analyses (relative risk, 5.9; 95% confidence interval [CI] 3.6 to 10.5). ^c^Fresh fruit included strawberries, raspberries, blueberries, and blackberries. Persons served themselves from a bowl of fresh strawberries and a bowl of fresh raspberries, blueberries, and blackberries next to the cake. The raspberries in the bowl came from a different source than the raspberries in the cake filling and were not statistically significant in multivariate analyses.

 We did not identify additional cases through the Pennsylvania State Bureau of Epidemiology’s alert to local health departments. Moreover, the catering company contacted the six groups to which they served raspberries during June 2–4, and no additional cases were identified.

### Laboratory Investigation

 For three of the five case-patients who had laboratory-confirmed cyclosporiasis (Table 1), stool specimens were sent to CDC and reconfirmed as positive for *Cyclospora* oocysts. Results of stool testing for other microbes were negative.

 PCR analyses conducted by both FDA and CDC confirmed the presence of *Cyclospora* DNA in the raspberry filling of the cake. At FDA, an FTA-based PCR identified *Cyclospora* DNA in the raspberry filling but not in the icing from the top of the cake or in the cake itself. At CDC, PCR amplification was possible from DNA directly extracted from four of the nine portions of the raspberry filling that were tested. All positive samples generated sequences that were identical to *C. cayetanensis* in the 636-bp PCR product.

### Environmental and Source Investigations

 None of the catering staff reported having been ill during the 3 weeks before or after the reception. However, on July 18, the local health department learned, through routine surveillance, about a caterer, who had worked during the reception, in whom gastrointestinal illness developed 8 days later; she had laboratory-confirmed cyclosporiasis. Although she could not recall her duties for the event, her usual tasks included cutting and serving wedding cake. She also identified two other staff members in whom gastrointestinal illness developed a median of 6 days after the reception; their symptoms resolved spontaneously, and no stool specimens were tested. They had not worked during the reception but had worked later that day and reported that they probably ate leftover cake.

**Figure 2:**
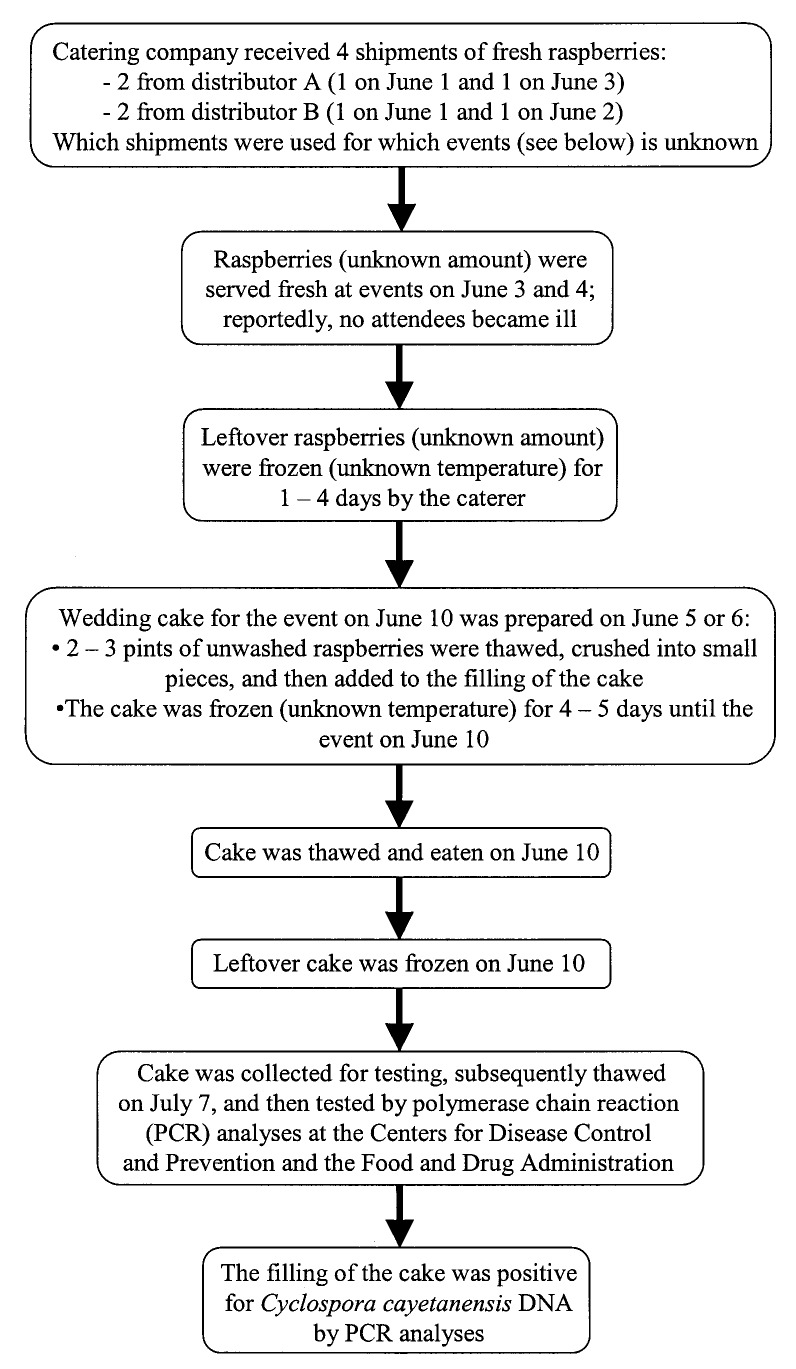
Flow chart with details about raspberries and wedding cake served at a wedding reception in June 2000 in Pennsylvania. The shipment of raspberries the catering company received on June 3 included raspberries from Guatemala. As noted, some details (e.g., the date the cake was prepared, the temperature of the freezer at the catering company) are unknown.

Raspberries were the only produce used in the wedding cake. The catering company had received four shipments of fresh raspberries June 1–3. Some of the raspberries from these shipments were served fresh at events on June 3–4. The leftover raspberries were frozen for 1–4 days. Two to three pints of leftover, unwashed raspberries were thawed, crushed into pieces, and folded into the butter cream filling used in the cake. The cake was then frozen for another 4–5 days until it was thawed and eaten on June 10, the date of the wedding (Figure 2).

**Figure 3:**
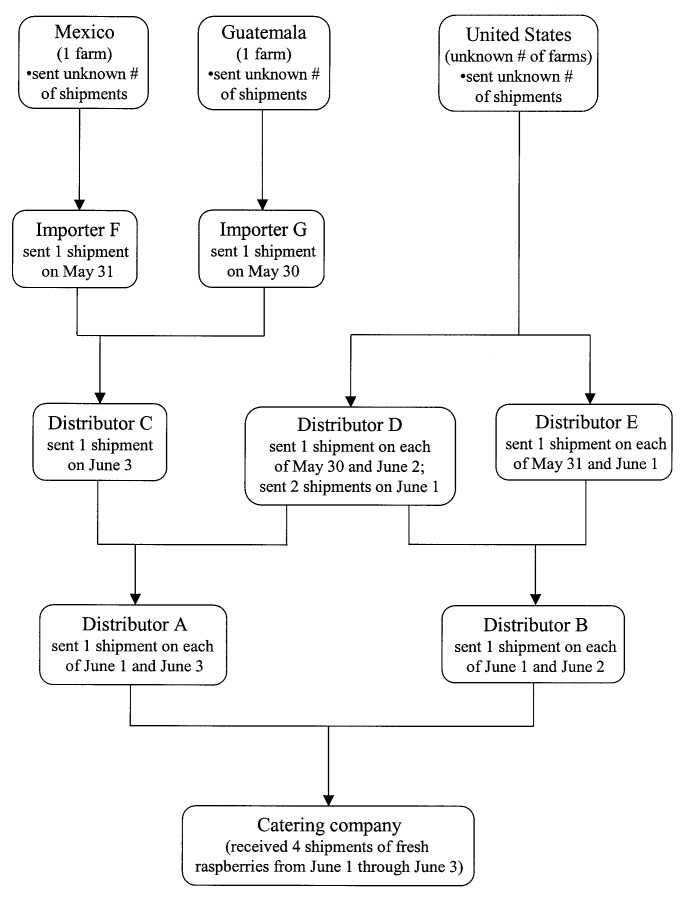
Flow chart with details about the possible sources of the raspberries used in the wedding cake served at a wedding reception in June 2000 in Pennsylvania. The Guatemalan farm was also a possible source of raspberries served at an event in Georgia in May 2000. This farm was the only known source in common to these two events.

 Local distributors A and B supplied the four shipments of raspberries received by the catering company during the period June 1–3 (Figure 3). Distributors A and B had received shipments from three other local distributors (C, D, and E) during May 30–June 3. During this period, one Guatemalan farm and one Mexican farm were the sources of the raspberries that were delivered to importers F and G that supplied distributor C, and an unknown number of U.S. farms supplied the raspberries delivered to distributors D and E (Figure 3). Which raspberries from which shipment were actually used in the cake is unknown.

 The one Guatemalan farm that could have supplied the raspberries used in the cake was also one of the possible sources (and the only Guatemalan source) of raspberries served at a bridal brunch in Georgia in May 2000 that was associated with an outbreak of cyclosporiasis (14). The Guatemalan farm was the only known source in common to the events in Pennsylvania and Georgia. FDA inspected this farm and found no definitive source for contamination (J. Guzewich, pers. comm).

## Discussion

We investigated an outbreak of cyclosporiasis associated with a wedding reception in Philadelphia in June 2000. The epidemiologic evidence and the identification by PCR analyses of *Cyclospora* DNA in the food item most strongly associated with illness indicated that the outbreak was foodborne. This is the first outbreak associated with raspberries for which *Cyclospora* DNA has been identified in the epidemiologically implicated food item. Because of the outbreak, the Philadelphia health department subsequently made cases of *Cyclospora* infection reportable.

We do not know whether the outbreak involved more cases in Pennsylvania than the cluster of cases we investigated. The catering company for the wedding reception received four shipments of fresh raspberries the weekend before the cake was prepared; which raspberries from which shipment were used for the reception rather than the other six events during the period of interest at which raspberries were served is unknown. We did not identify any more cases of cyclosporiasis by contacting the other six groups. However, perhaps only one of the shipments of raspberries and only some of the raspberries in that shipment were contaminated. The same Guatemalan farm was one of the possible sources of the raspberries (and the only Guatemalan source) served at the reception in Pennsylvania in June 2000 and the bridal brunch in Georgia in May 2000. The identification of only one farm in common to the two events suggests that the events were related.

The year 2000 was the fifth year since 1995 (i.e., 1995, 1996, 1997, 1998, and 2000) that outbreaks of cyclosporiasis occurred in the spring in the United States or Canada that definitely or probably were associated with Guatemalan raspberries (7). However, the recent outbreaks have been much smaller than the multistate outbreaks in 1996 and 1997 (7). After the outbreaks in 1996 and 1997 (2,3), FDA began working with the Guatemalan government and berry industry to improve farming and exporting practices for raspberries. Only farms that meet certain standards—including water, sanitation, and worker hygiene issues—have been allowed to export fresh raspberries to the United States during the “spring season” (March through August). The standards are reviewed and updated yearly. During the spring of 2000, five Guatemalan farms were allowed to export to the United States. After the outbreaks in Pennsylvania and Georgia, FDA did not allow the farm that was in common to the events to export raspberries to the United States during the spring of 2001. No U.S. outbreaks of cyclosporiasis associated with Guatemalan raspberries were identified that spring. During the spring of 2002, only three farms, which have never been implicated in outbreaks of cyclosporiasis, were allowed to export raspberries to the United States.

The cyclosporiasis outbreak in Philadelphia is the first for which raspberries that reportedly had been frozen, rather than served fresh, were implicated. The raspberries used in the wedding cake had been bought fresh but then reportedly frozen and thawed twice before they were served (Figure 2). The temperature of the freezer where the raspberries were stored is unknown. When the freezer was inspected 2 weeks after the outbreak, the temperature of the freezer was -3.3°C, which might not have been cold enough to kill *Cyclospora* oocysts. Although limited information is available about the effects of freezing on their survival, for *Cryptosporidium parvum*, another coccidian parasite, oocysts remain infectious for mice after storage at –10°C for 1 week (15). Freezing at sufficiently cold temperatures for sufficiently long periods, as during commercial freezing, should kill *Cyclospora* oocysts. For *Crytosporidium parvum,* oocysts were not infectious for mice after storage at –15°C for 1 week (15).

This Pennsylvania outbreak is the second documented U.S. outbreak, and the first associated with raspberries, for which *Cyclospora* has been detected in the epidemiologically implicated food item. The first U.S. outbreak for which *Cyclospora* was detected in a food item occurred in 1999 in Missouri and was associated with fresh basil in a chicken pasta salad (8). Neither the raspberries in 2000 nor the basil in 1999 had been washed. Although all produce should be thoroughly washed before being eaten, other outbreaks (2,3,7) and a laboratory study (16) have shown that washing does not eliminate the risk for *Cyclospora* infection.

Fortunately, leftovers from both the Pennsylvania and Missouri outbreaks had been frozen and were available for testing. Contamination of the implicated food items for the 1999 outbreak was demonstrated by PCR analyses and light microscopy and for the 2000 outbreak by PCR analyses. The modes of contamination of the produce associated with outbreaks of cyclosporiasis have not yet been definitively identified for any of the outbreaks. However, molecular methods are sensitive tools that can be used to identify the organism in the vehicle and in environmental samples, which then may provide information useful for determining the mode of contamination. Molecular methods for detecting *Cyclospora* continue to improve (9). Continued research about the biology and epidemiology of *Cyclospora* is important for identifying effective measures for preventing future outbreaks.
